# Anti-interleukin 1 treatment in secondary renal amyloidosis associated with autoinflammatory diseases

**DOI:** 10.1186/1546-0096-13-S1-P149

**Published:** 2015-09-28

**Authors:** R Topaloglu, ED Batu, D Orhan, S Ozen, N Besbas

**Affiliations:** 1 Hacettepe University Faculty of Medicine, Department of Pediatrics, Division of Nephrology, Ankara, Turkey; 2 Hacettepe University Faculty of Medicine, Department of Pediatrics, Division of Rheumatology, Ankara, Turkey; 3 Hacettepe University Faculty of Medicine, Department of Pediatric Pathology, Ankara, Turkey

## Introduction

Amyloidosis represents a heterogeneous group of disorders characterized by extracellular deposition of autologous fibrillary proteins which impair normal organ function. Reactive AA type amyloidosis may complicate autoinflammatory diseases (AID).

## Objective

To evaluate and compare the renal biopsy findings and clinical and laboratory parameters in patients with amyloidosis secondary to AID who have responded to the anti-interleukin 1 (IL1) treatment.

## Patients and methods

Two children with systemic juvenile idiopathic arthritis and one with cryopyrin-associated periodic syndrome diagnosed as AA type amyloidosis were treated with anti-IL1 drugs and we have evaluated the course and management of these patients for a follow-up of median 56 (41-56) months. The renal biopsies at the time of diagnosis of amyloidosis and after the onset of anti-IL1 treatment were evaluated and compared according to the amyloid scoring and grading system based on the histopathological findings.

## Results

The median age of AID onset was three years, while the patients were diagnosed to have amyloidosis at a median of 12 years of age. The patients previously used nonsteroidal anti-inflammatory drugs, corticosteroid, methotrexate, azathioprine, infliximab, and intravenous immunoglobulin treatments. After the diagnosis of amyloidosis, anakinra was started. All three responded to anakinra treatment; however, canakinumab was commenced in patient 3 since anakinra caused local cutaneous reaction at the site of drug administration. Proteinuria was improved in patients after anti-IL 1 treatment. Control renal biopsies were performed a median of three years later than the diagnosis of amyloidosis. At the renal biopsy level, we have seen that the renal amyloid prognostic score did not improve in patient 1 and progressed in patient 2 and 3. The renal amyloid grade has also progressed in patient 2.

## Conclusion

To the best of our knowledge, this is the first series showing progression of renal tissue damage after the improvement of proteinuria with anti-IL 1 treatment in AID-associated amyloidosis. After the development of amyloidosis, it is crucial to control inflammation effectively and prevent further amyloid accumulation in patients with anti-inflammatory treatments such as anti-IL1 drugs. However, new treatment strategies are needed to target the amyloid deposits for patients with severe organ involvement.

